# Role of the G Protein-Coupled Receptors in Cancer and Stromal Cells: From Functions to Novel Therapeutic Perspectives

**DOI:** 10.3390/cells12040626

**Published:** 2023-02-15

**Authors:** Rosamaria Lappano, Marcello Maggiolini

**Affiliations:** Department of Pharmacy, Health and Nutritional Sciences, University of Calabria, 87036 Rende, Italy

G protein-coupled receptors (GPCRs) are transmembrane signal transducers that regulate a plethora of physiological and pathological processes. As such, they represent one of the most important attractive therapeutic targets [1]. Coupling with G proteins, GPCRs transmit numerous extracellular signals generated by hormones, neurotransmitters, ions, photons, odorants and chemokines, among others [2]. In particular, agonist-bound GPCRs interact with heterotrimeric G proteins, which consist of α, β and γ subunits, inducing the exchange of guanosine diphosphate (GDP) for guanosine triphosphate (GTP) on the Gα subunit. Gα then dissociates from Gβγ transducing signals to diverse downstream effectors, which include calcium, adenylyl cyclase, phospholipase C, protein kinases and small GTP-binding proteins, depending on which of the four Gα families (Gαs, Gαi, Gαq, and Gα12/13) has been engaged [2]. In contrast to initial hypotheses sustaining a role for Gβγ exclusively in the inactivation of Gα subunit and the enhancement of membrane binding [3], Gβγ has been widely shown to represent an essential contributor to GPCR signaling pathways given that it can directly regulate many different downstream targets [4]. In addition to G proteins, GPCRs can recruit G protein-coupled receptor kinases (GRKs) and arrestins, which are known to be implicated in both the desensitization and responsiveness of GPCRs [5]. In particular, GRK-mediated GPCR phosphorylation promotes the binding of β-arrestins, which play a role not only in uncoupling receptors from G proteins but also act as adaptors for agonist-induced GPCR endocytosis and kinase activation toward the activation of signaling pathways within endosomes [6]. Considering that GPCRs are involved in multiple cellular processes by engaging diverse signal transducers in response to different ligands, it is not surprising that they participate in the control of almost all physiological functions, including neurotransmission, cardiovascular, skeletal, respiratory and immune regulation [7]. Consequently, changes in GPCR levels, subcellular location and the extent of activation may contribute to the initiation of various pathogenic processes, including cancer development. Indeed, GPCRs mutation, aberrant expression or hyperactivation, along with the increased release of their agonists, are accounted as the main molecular routes through which they can act as cancer drivers [8,9]. In particular, GPCRs may function as pro-tumorigenic signaling hubs by participating in cell growth and survival, invasion and metastasis, as well as angiogenesis, tumor-promoting inflammation and immune evasion [9]. Moreover, GPCRs, their agonists, heterotrimeric G proteins and diverse downstream effectors can contribute to generating a tumor-promoting microenvironment (TME), therefore supporting cancer progression [10,11,12,13,14]. In particular, TME consists of a wide variety of cell types, such as fibroblasts, endothelial, immune and inflammatory cells, which are embedded in modified non-cellular components of the extracellular matrix, for instance, collagen, fibronectin and laminin [15]. Of note, GPCRs and their cognate ligands directly stimulate a pro-tumorigenic TME and also behave as signaling network hubs that harmonize the bi-directional liaison between cancer and stromal cells within the TME [8,16,17,18,19]. Based on the aforementioned findings, a crucial role for the stromal components of TME has been broadly recognized during cancer initiation and metastatic spread, thus suggesting the development of a broad spectrum of TME-targeted therapies [15,20].

This Special Issue aims to provide an attractive assortment of novel research on the molecular routes and the cellular events engaged by GPCRs to function as pivotal oncogenic drivers. Moreover, the papers presented focus on offering advanced knowledge regarding the therapeutic value of targeting GPCRs as attractive candidates for anti-cancer treatments.

The first group of articles focuses on the role of a GPCR, namely the G protein-coupled estrogen receptor (GPER) in TME [14,21,22]. The GPER is a seven-transmembrane protein that mediates rapid estrogen signaling in both normal and neoplastic cells [14]. Similar to other GPCRs, GPER activation triggers the transactivation of the epidermal growth factor receptor (EGFR) through the cleavage of the heparin-bound EGF on the plasma membrane, leading to downstream signaling events, such as calcium mobilization, cyclic adenosine monophosphate (cAMP) synthesis and the activation of diverse kinase pathways [23]. Engaging these signaling mediators, the GPER can also regulate the expression of certain genes, which are implicated in many cancer hallmarks, including sustained proliferation, invasion and metastasis and angiogenesis and tumor-promoting inflammation [14]. As underlined by Papermans et al., GPERs may play a role in cancer cells and in various stromal cells within the TME named cancer-associated fibroblasts (CAFs), immune cells and adipocytes [14]. From this perspective, the authors reviewed previous evidence showing that CAFs can induce paracrine signals toward a pro-angiogenic TME and promote breast tumor migration and invasion through GPER [14]. The clinical significance of the GPER has been explored in a wide variety of malignancies, particularly breast, ovarian and endometrial cancers, wherein the levels of GPER are directly related to the poorer survival of patients [24]. Regarding breast cancer, immunohistochemical studies revealed that GPER expression is associated with increased tumor size, distant metastasis and recurrence [25,26]. Moreover, both clinical and experimental studies have also indicated that the GPER is a potential player in resistance to endocrine therapy [27,28]. Next, considering that the GPER also mediates the stimulatory effects of estrogens in breast cancers that lack the classical estrogen receptor (ER) [29,30], Talia et al. took advantage of the information provided by The Invasive Breast Cancer Cohort of The Cancer Genome Atlas (TCGA) project and Molecular Taxonomy of Breast Cancer International Consortium (METABRIC) datasets in order to assess the biological significance of GPER in ER-negative breast tumors [31]. Bioinformatics analyses revealed that GPER expression is associated with pro-migratory and metastatic genes belonging to cell adhesion molecules (CAMs), extracellular matrix (ECM)-receptor interaction and focal adhesion (FA) signaling pathways, in agreement with previous evidence, indicating the potential of GPER to contribute to the spread and metastatic outgrowth of ER-negative breast cancer cells [32,33]. In addition, a shorter disease-free interval (DFI) has been shown in ER-negative BC patients with high GPER expression with respect to patients characterized by low levels of GPER [31]. Based on the acknowledged role exerted by GPER in cancer and other diseases [14,34], this receptor has been suggested as a promising therapeutic target for the treatment of obesity, diabetes and vascular pathology. In this regard, the potential value of targeting GPER in combination with immune checkpoint inhibitors, particularly in melanoma, has also been discussed by Papermans et al. [14]. In addition to ligands that selectively target GPER, a peptide corresponding to the 295-311 fragment of the hinge/AF2 domains of the human ERα (ERα17p) has been characterized as a GPER inverse agonist [35]. By triggering a proteasome-dependent decrease in GPER levels, ERα17p leads to the inhibition of GPER-mediated signaling toward anti-proliferative responses in breast cancer cells [35]. Of note, Trichet et al. provided novel insights into the mechanisms of action of ERα17p, including its direct membrane effects [22]. In agreement with previous findings showing that ERα17p interacts with membrane models [35], far-UV circular dichroism (CD), plasmon waveguide resonance (PWR) and fluorescence (leakage assay) spectroscopy techniques, ERα17p has been shown to interact with lipid bilayers mimicking the eukaryotic plasma membranes, as observed using breast cancer cells [22].

Several studies have investigated whether GPER may also play a role in testicular, prostate, thyroid and lung cancers [21,36,37,38]. In this respect, Chimento et al. focused on the physiological regulation of testicular function by GPER and its involvement in the neoplastic transformation of both germ and somatic cells [21]. GPER is overexpressed in ERα-negative testicular seminoma and embryonal cancerous tissues and cells, wherein it mediates proliferative effects in response to estrogens [21]. On the contrary, in Leydig cell tumors expressing ERα, the activation of GPER triggers reduced cell growth [21]. Moreover, Chimento et al. highlighted the functional interaction among GPER and nuclear receptors, namely ERs, peroxisome proliferator-activated receptors (PPARs) and estrogen-related receptors (ERRs) in both interstitial and tubular compartments. For instance, the cooperation between GPER and PPARs is involved in the secretion of estrogen and progesterone and in regulating lipid metabolism and steroidogenesis in Leydig cell tumors [21]. These observations indicate that GPER could be considered to be a promising target together with certain nuclear receptors for the assessment of new pharmacological approaches in estrogen-sensitive testicular tumors. Likewise, the above-mentioned evidence might pave the way for new perspectives to better understand the homeostasis and metabolism of lipids within the testis and, therefore, their role in testicular tumorigenesis.

The thyroid-stimulating hormone (TSH) receptor (TSHR) is a hormone GPCR belonging to the leucine-rich repeat (LGR) subfamily [39]. TSHR is predominantly expressed in thyroid tissues, where it serves as a target for TSH, regulating thyrocyte differentiation and growth and thyroid hormone synthesis [39]. Additionally, TSHR and its downstream effectors are considered to be the main players in the development and progression of thyroid cancer [40]. Likewise, the expression of TSHR has been determined in ovarian and liver cancer, suggesting its role in further types of tumors [40]. The clinical usefulness of targeting TSHR-activated pathways in anti-cancer therapies has been summarized by Chu et al. [41]. In particular, the suppression of TSH by the administration of an artificially synthesized mimic of thyroxine, namely levothyroxine, represents a mainstay of clinical management for patients affected by differentiated thyroid cancer, especially for diseases with a high risk of recurrence [41]. However, the adverse effects caused by subclinical hyperthyroidism and the small benefits for patients with a low risk of recurrence may suggest the need for more individualized strategies for treating thyroid cancer, such as novel TSHR antagonists and TSH-coated nanoliposomes packed with chemotherapeutics [41].

The renin–angiotensin system (RAS) is a network of GPCRs (i.e., AT1R, AT2R, MASR and MRGD) and peptide hormones (i.e., angiotensin II, angiotensin 1–7 and alamandine), which are produced by angiotensin-converting enzyme (ACE) [42]. The RAS system plays a regulatory role in many aspects of human physiology, including the cardiovascular, pulmonary, renal and immune systems [43]. The dysregulation of the RAS-mediated endocrine balance is implicated in cardiovascular pathologies and in different diseases, including cancer [43]. In this regard, Acconcia recapitulated the recent knowledge on angiotensin receptors in breast cancer while also discussing the anti-neoplastic potential of targeting angiotensin receptors [44]. In particular, the preclinical studies discussed may provide a rationale for the potential use of angiotensin receptor blockers in breast cancer patients [44]. Furthermore, data regarding the contribution of the Mas-related GPCR member D (MRGD) to the growth of breast cancer cells and the resistance to endocrine therapy have been discussed, pointing out that diverse GPCR members of the RAS system may offer multifaceted opportunities for the development of novel therapies for the treatment of breast tumors [44].

Metabolite-sensing GPCRs belong to a newly characterized class of GPCRs, which are selectively activated by different metabolites, such as fatty acids, hydroxycarboxylic acids, bile acids, amino acids, protons and the Krebs cycle intermediate succinate. Metabolite-sensing GPCRs regulate not only the metabolism but also immunity and inflammation, therefore providing a further connection between the immune and metabolic systems [45]. Considering the metabolic alterations that characterize tumor cells and the main role that inflammatory responses elicited in tumor initiation and progression, it is no wonder that the action of certain metabolite-sensing GPCRs may be of particular relevance in tumor cells [46]. In this perspective, Cosín-Roger et al. outlined recent evidence linking metabolite-sensing GPCRs to cancer progression [46]. The review clearly pointed out that this field needs thorough and large-scale clinical investigations to better define the biological value of these receptors in diverse types of malignancies. Notably, most metabolite-sensing GPCRs may exhibit pro- or anti-tumorigenic effects depending on the tumor context [46]. Nevertheless, a differential expression of these receptors is shown in several human tumors compared with the respective normal tissues, suggesting their role as potential cancer tissue-specific biomarkers [46].

We sincerely hope that the papers collected in this Special Issue of *Cells* might encourage further acknowledgment regarding the impact of GPCRs in cancer cells and the components of the surrounding microenvironment toward the development of personalized therapeutic tools targeting GPCRs and their downstream signaling effectors.
